# Gut microbiome alpha-diversity is not a marker of Parkinson’s disease and multiple sclerosis

**DOI:** 10.1093/braincomms/fcab113

**Published:** 2021-06-01

**Authors:** Jonathan Plassais, Guillaume Gbikpi-Benissan, Marine Figarol, Filip Scheperjans, Guy Gorochov, Pascal Derkinderen, Alessandra C L Cervino

**Affiliations:** Luxia Scientific, HCenter, 77000 La Rochette, France; STAT-ALLIANCE, 92700 Colombes, France; Luxia Scientific, HCenter, 77000 La Rochette, France; Luxia Scientific, HCenter, 77000 La Rochette, France; Department of Neurology, Helsinki University Hospital, Helsinki 00290, Finland; Department of Clinical Neurosciences (Neurology), University of Helsinki, Helsinki 00014, Finland; Sorbonne Université, Inserm, Centre d’Immunologie et des Maladies Infectieuses (CIMI-Paris), 75013 Paris, France; CHU de Nantes, Hôpital Laennec, 44800 Saint Herblain, France; UMR Inserm 1235, Faculté de Médecine, 44035 Nantes, France; Luxia Scientific, HCenter, 77000 La Rochette, France

**Keywords:** Parkinson’s disease, multiple sclerosis, alpha-diversity, meta-analysis, 16S rRNA gene amplicon sequencing

## Abstract

The gut–brain axis may play a central role in the pathogenesis of neurological disorders. Dozens of case–control studies have been carried out to identify bacterial markers by the use of targeted metagenomics. Alterations of several taxonomic profiles have been confirmed across several populations, however, no consensus has been made regarding alpha-diversity. A recent publication has described and validated a novel method based on richness and evenness measures of the gut microbiome in order to reduce the complexity and multiplicity of alpha-diversity indices. We used these recently described richness and evenness composite measures to investigate the potential link between gut microbiome alpha-diversity and neurological disorders and to determine to what extent it could be used as a marker to diagnose neurological disorders from stool samples. We performed an exhaustive review of the literature to identify original published clinical studies including 16S rRNA gene sequencing on Parkinson’s disease, multiple Sclerosis and Alzheimer’s disease. Richness and evenness factors loadings were quantified from sequencing files in addition with the Shannon diversity index. For each disease, we performed a meta-analysis comparing the indices between patients and healthy controls. Seven studies were meta-analysed for Parkinson’s disease, corresponding to 1067 subjects (631 Parkinson’s Disease/436 healthy controls). Five studies were meta-analysed for multiple sclerosis, corresponding to 303 subjects (164 Multiple Sclerosis/139 healthy controls). For Alzheimer’s disease, the meta-analysis was not done as only two studies matched our criteria. Neither richness nor evenness was significantly altered in Parkinson’s disease and multiple sclerosis patients in comparison to healthy controls (*P*-value > 0.05). Shannon index was neither associated with neurological disorders (*P*-value > 0.05). After adjusting for age and sex, none of the alpha-diversity measures were associated with Parkinson’s Disease. This is the first report investigating systematically alpha-diversity and its potential link to neurological disorders. Our study has demonstrated that unlike in other gastro-intestinal, immune and metabolic disorders, loss of bacterial diversity is not associated with Parkinson’s disease and multiple sclerosis.

## Teaser

Plassais et al. report a meta-analysis of gut microbiome alpha-diversity which demonstrates that richness and evenness were altered neither in Parkinson’s disease patients nor in Multiple Sclerosis patients in comparison with controls. This is the first report investigating systematically alpha-diversity and its potential link to neurological disorders.

## Graphical Abstract

**Graphical Abstract:**
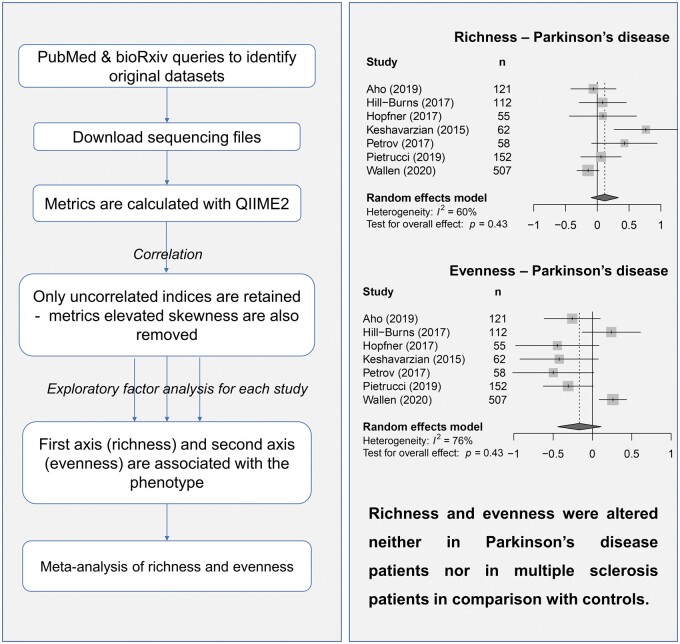

## Introduction

There is mounting evidence that gut microbiota plays a central role in the development and prognosis of neurological disorders (ND).^1^^,^^2^ The bidirectional communication pathway between gut bacteria and the central nervous system is now referred to as the microbiota–gut–brain axis.^3–5^ The prototypical gut–brain disorder is Parkinson’s disease.^6^ Gastro-intestinal (GI) symptoms occur in almost every Parkinson’s disease patient at some point^7^ and autopsy studies have consistently shown that alpha-synuclein aggregates, the defining neuropathological hallmark of the disease, are found in the gut in nearly every case.^8^^,^^9^ For Multiple Sclerosis (MS), nearly two-thirds of patients exhibit at least one persistent GI symptom in the disease course^10^ and recent reports suggested that the GI tract and especially the enteric nervous system were targeted by the autoimmune process in both experimental and human MS.^11^^,^^12^ Regarding Alzheimer’s disease, emerging evidence also suggests the existence of GI comorbidities and a preliminary report showed the presence of histological changes in the gut of Alzheimer’s disease subjects.^13^

Microbial communities that colonize our gut can be studied in a culture-independent manner by the use of new sequencing technologies, and particularly the widely used 16S rRNA gene sequencing.^14^^,^^15^ Numerous clinical cross-sectional observational studies have highlighted the association between ND and gut bacterial composition.^16–43^ All of these studies have reported shifts in abundance of bacteria by comparing gut microbiota of ND patients to gut microbiota of healthy donors. Recently, a meta-analysis at family and genus levels has helped in confirming bacterial changes between Parkinson’s disease subjects and aged-match donors.^44^ No meta-analysis on the gut microbiota composition in MS and Alzheimer’s disease are available. And as far as we know, bacterial diversity has not been exhaustively investigated in ND.

Alpha-diversity (α-diversity) is a numeric value summarizing the structure ecological community, for a single metagenomic sample, with respect to its richness, evenness or both.^45^ Richness commonly refers to the number of unique species that are present within a sample, while evenness refers to how species are held in even abundance with each other within a sample.^46^ Recently, Hagerty et al. have proposed an empirically derived method for measuring α-diversity, reducing all uncorrelated indexes to simple richness and evenness factors. The use of composite measures aims at reducing measurement error and increasing reliability, and the standardization step resulted on composite scores on the same scale between studies. Hence given the contradictory and conflicting results that had been obtained on gut microbiome α-diversity in ND, we set out the current research to meta-analyse both richness and evenness composite measures for each ND.

## Materials and methods

### Identification of original studies

After a first review of the literature, we focussed our research of original published data on Parkinson’s Disease, Multiple Sclerosis and Alzheimer’s disease, the three most studied diseases for their association with gut microbiota. Then, we performed three distinct queries using the PubMed database: Query#1 “Parkinson*[Title/Abstract] AND (Microbio*[Title/Abstract] OR Dysbiosis[Title/Abstract])”, Query#2 “Multiple Sclerosis [Title/Abstract] AND (Microbio*[Title/Abstract] OR Dysbiosis[Title/Abstract])” and Query#3 “Alzheimer*[Title/Abstract] AND (Microbio*[Title/Abstract] OR Dysbiosis[Title/Abstract])”.

After exclusion of review articles, we reviewed all titles and/or abstracts and we included publications based on the following criteria:

- Parkinson, MS or Alzheimer is the phenotype of interest
- Human case–control studies, on adults only (≥18 years)
- Analysis of gut microbiome using 16S ribosomal RNA gene sequencing on Illumina platform
- Availability of full microbiome data (fastq files)

Owing to the low number of studies available for Alzheimer’s disease, we decided to focus our analysis on Parkinson and MS only. Authors were contacted when metagenomic data were not publicly available or to have access to metadata and/or clinical data.

### Bioinformatics processing

Fastq files from each study were downloaded from public databases (i.e. the Sequencing Read Archive database) and when datasets were not publicly available, we requested access from authors. Non-faecal samples were excluded. For each study, fastq files were processed using the QIIME2™ pipeline (version 2019.10)^47^ and the quality of raw sequencing reads was assessed with FastqC. Reads were quality filtered, chimera-checked and clustered in amplicon sequencing variants using Deblur. The taxonomy of representative sequences was assessed using the RDP database (version 11).^48^

Alpha-diversity indices were computed in the QIIME2™ pipeline. We considered the nine following indices as reported in Hagerty et al. 2020: Menhinick, Fisher alpha, Faith pd, Shannon, Lladser pe, ENSpie (equivalent to the inverse Simpson index), Strong, Heip e and Simpson evenness measure E (Simpson e). Rarefaction curves were drawn, and each diversity index was estimated by rarefying samples at 10 000 reads.

### Statistical analysis

The statistical analysis was performed using R (version 3.6.3). R scripts were compiled using the knitr package and saved in HTML. We performed a two-step statistical analysis. Firstly, richness and evenness were estimated from each study as suggested by Hagerty et al.^46^ Briefly, for each original study, all α-diversity measures were loaded and then we performed an exploratory factor analysis (EFA) using the package *psych* (v. 1.9.12). From each EFA, we obtained richness and evenness measures, two composite measures and orthogonal to one another, resulting from the two first factors. Before performing the meta-analysis, we checked for consistency between studies (correlation between α-diversity measures, correlation of α-diversity measures with the two composite measures, orthogonality between richness and evenness).

Next, for each disease (Parkinson’s disease and MS), we performed a meta-analysis of richness and evenness. Linear regressions were fitted to estimate standardized mean differences (SMD) for each diversity measure, with or without adjustment for cofounding factors (age and sex). Then, overall estimates were estimated by doing a weighting inverse variance meta-analysis with a random model. The generic function metagen from the R package meta (version 4.12) was used. The overall estimate and its 95% confidence interval were reported, in addition to *P*-value calculated based on the *t* distribution. *P*-values were adjusted using Benjamini–Hochberg correction. For Parkinson’s disease studies, adjustment for confounding factors was limited to age and sex as no other clinical variable was available. For MS, clinical data were available for only one study, hence no adjustment was possible.

### Data availability statement

Almost all datasets used for this manuscript (16S rRNA sequences) are publicly available, with exception for Petrov et al.,^18^ Chen et al.^26^ and Cekanaviciute et al.^25^ Clinical data and metadata are not publicly available and cannot be shared for restriction with authors. An HTML file with R code will be available under request.

## Results

### Identification and selection of studies

**Figure 1:**
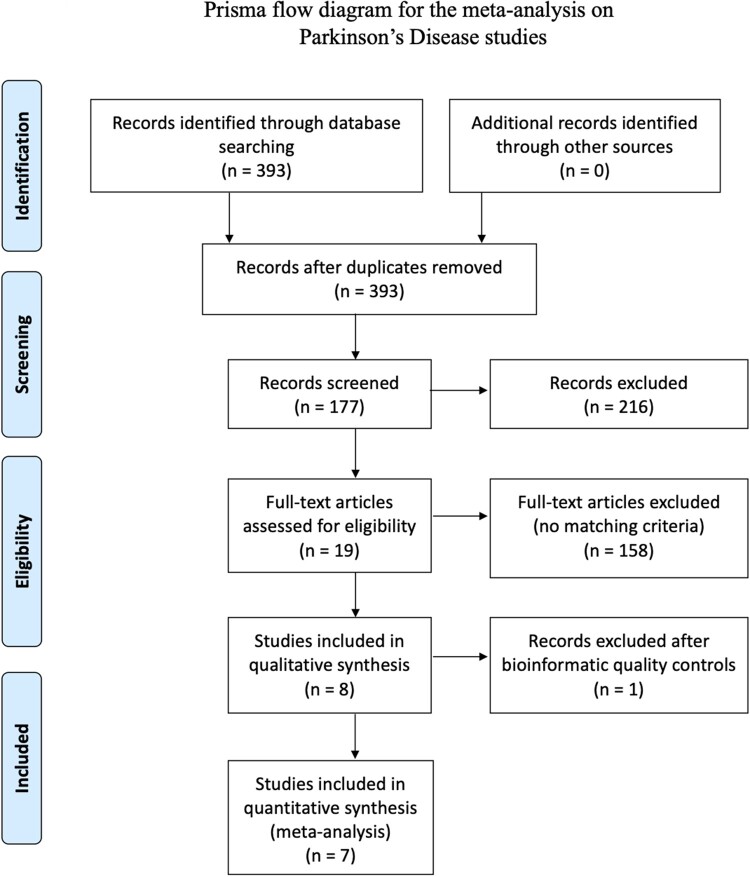
PRISMA flowchart for meta-analysis on Parkinson’s disease.

**Figure 2:**
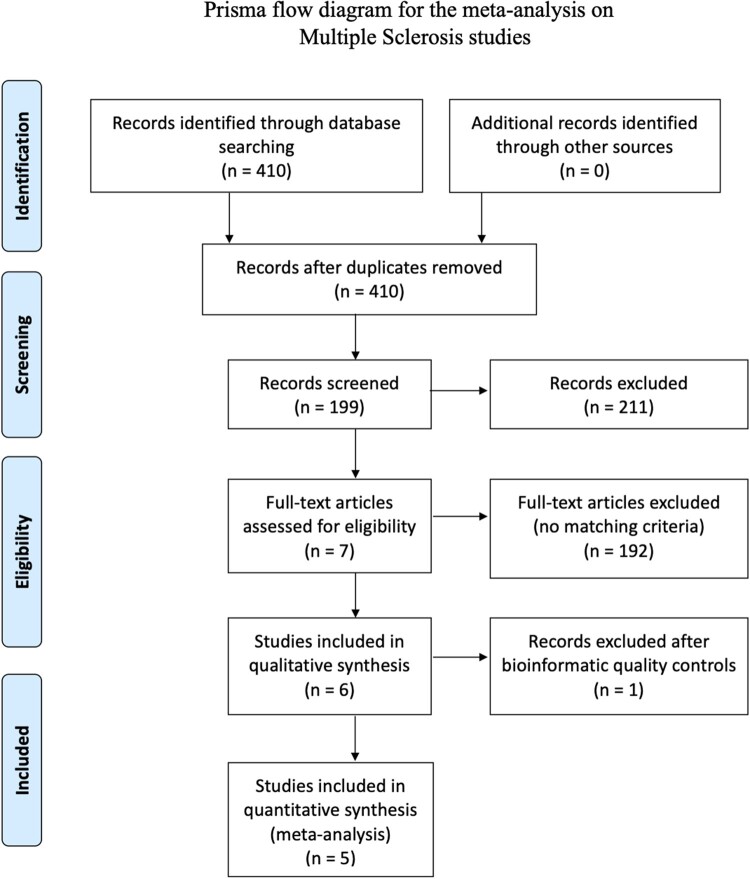
PRISMA flowchart for meta-analysis on Multiple Sclerosis.

On 30 June 2020, a total of 393 records were identified from PubMed for Parkinson’s Disease (Query#1), 410 records for Multiple Sclerosis (Query#2) and 432 records for Alzheimer’s Disease (Query#3) (Supplementary Tables 1–3). PRISMA flow chart was done for Parkinson’s disease (see Fig. 1) and MS (see Fig. 2). After removing review articles, we reviewed all titles and/or abstracts. Nineteen studies^16–20^^,^^22^^,^^23^^,^^28^^,^^36–43^^,^^49–51^ with original gut microbiome data were found for Query#1, seven studies^25–27^^,^^29^^,^^30^^,^^52^^,^^53^ for Query#2 and only two studies^34^^,^^35^ for Query#3. Sequencing metagenomic data, clinical data and metadata were not publicly available for all studies. Authors were contacted for providing raw sequencing files and/or limited clinical data from which age, gender, BMI or weight, and constipation status. Finally, we had access to metagenomic datasets for ten original articles^16^^,^^18–20^^,^^23^^,^^36–40^ where Parkinson’s disease was the main phenotype of interest and six original articles^25–27^^,^^29^^,^^30^^,^^52^ for MS. For two studies^19^^,^^20^ we were not able to associate sequencing files to the clinical phenotype, hence these two studies were discarded. Moreover, clinical data (at least age and gender) were available for five Parkinson’s disease studies only.^16^^,^^18^^,^^23^^,^^38^^,^^40^ We had no access to raw metagenomic sequencing files for Alzheimer’s disease, hence we did not perform the meta-analysis for this disease.

### Sample processing

We processed all samples with our internal bioinformatics pipeline (see Methods). We compared per-sample sequence counts between studies after quality controls (Supplementary Fig. 1). For both Parkinson’s disease and MS studies, the number of sequences was highly variable within and between each study. We defined a threshold at 10 000 sequences to ensure enough accuracy of α-diversity estimates and comparability between samples and studies. Four studies reached this threshold for all samples, but two studies, Heintz-Buschard et al. and Miyake et al., did not reach the threshold for any of these samples while up to 61% of samples were removed for others. Therefore, these two studies were discarded, and the meta-analysis on Parkinson’s disease studies included a total of 1067 samples (631 Parkinson’s disease and 436 HC) and the meta-analysis on MS studies included a total of 303 samples (164 MS and 139 HC).

### Richness and evenness, two factors summarizing α-diversity indices

Nine diversity indices were estimated using QIIME2. For each study, we performed an EFA to estimate richness and evenness. We first confirmed that the two first factors explained the largest amount of variance (over 88% for all studies). One factor was highly correlated to Menhinick, Faith pd and Fisher alpha with Pearson’s correlation coefficients over 0.90 (Table 1 for Parkinson’s disease studies, Table 2 for MS studies). This factor loading was also correlated to the Llader pe index, however, the correlation was lower (an average of 0.63 for Parkinson’s disease studies, 0.60 for MS studies). Consequently, it was associated to richness as Menhinick, Faith pd and Fisher alpha are commonly associated to the number of species, and so to the richness. The other factor loading was highly correlated to Simpson e, Heip e, ENSpie and Strong with Pearson’s correlation coefficients over 0.75 (Tables 1 and 2), and it was associated to evenness. When one α-diversity index was correlated to the richness, it was not correlated to the evenness, and vice versa. This was due to the orthogonality between these two factors. Interestingly, this was not the case for the Shannon index, where its correlation with richness and evenness was between 0.58 and 0.71 (Pearson’s coefficient, see Tables 1 and 2). In light of these results and given the popularity of the Shannon index, we also performed the meta-analysis for the Shannon index.

**Table 1:** Correlation between α-diversity measures and Richness/Evenness estimated using an Exploratory Factor Analysis on Parkinson’s disease studies

Variables	Aho**^16^**		Hill-Burns**^38^**		Hopfner**^39^**		Keshavarzian**^40^**		Petrov**^18^**		Pietrucci**^36^**		Wallen**^23^**		Average	
	Richness	Evenness	Richness	Evenness	Richness	Evenness	Richness	Evenness	Richness	Evenness	Richness	Evenness	Richness	Evenness	Richness	Evenness
Richness	1.00	0.01	1.00	−0.02	1.00	0.01	1.00	−0.03	1.00	0.01	1.00	−0.04	1.00	0.00	–	−0.01
Evenness	0.01	1.00	−0.02	1.00	0.01	1.00	−0.03	1.00	0.01	1.00	−0.04	1.00	0.00	1.00	−0.01	–
Fisher alpha	0.99	0.15	0.96	0.22	0.98	−0.02	0.97	0.20	0.99	0.12	0.98	0.15	0.96	0.25	0.97	0.15
Menhinick	0.99	0.14	0.96	0.22	0.99	−0.04	0.97	0.20	0.99	0.12	0.98	0.15	0.96	0.25	0.98	0.15
Faith pd	0.86	0.22	0.94	0.17	0.84	0.14	0.95	0.09	0.94	0.00	0.95	0.10	0.88	0.24	0.91	0.14
Lladser_pe	0.70	−0.08	0.64	0.05	0.60	−0.39	0.52	0.06	0.58	0.00	0.70	−0.11	0.70	−0.05	0.63	−0.07
Heip e	−0.01	0.96	0.16	0.93	0.01	0.93	0.09	0.94	−0.10	0.94	−0.01	0.95	0.11	0.97	0.04	0.94
Simpson e	−0.23	0.90	−0.12	0.88	−0.04	0.87	−0.11	0.89	−0.25	0.87	−0.15	0.87	−0.12	0.91	−0.15	0.88
ENSpie	0.28	0.88	0.41	0.78	0.62	0.63	0.51	0.75	0.32	0.83	0.48	0.76	0.39	0.81	0.43	0.78
Strong	0.31	0.82	0.45	0.69	0.03	0.80	0.30	0.72	0.14	0.76	0.11	0.82	0.30	0.79	0.23	0.77
Shannon	0.61	0.75	0.66	0.67	0.77	0.51	0.65	0.69	0.56	0.76	0.66	0.68	0.58	0.75	0.64	0.69

**Table 2:** Correlation between α-diversity measures and Richness/Evenness estimated using an Exploratory Factor Analysis on MS studies

Variables	Cekanavicuite^25^		Chen^26^		Forbes^27^		Jangi^52^		Kozhieva^29^		Average	
	Richness	Evenness	Richness	Evenness	Richness	Evenness	Richness	Evenness	Richness	Evenness	Richness	Evenness
Richness	1.00	−0.01	1.00	−0.05	1.00	−0.05	1.00	−0.01	1.00	−0.02	–	−0.03
Evenness	−0.01	1.00	−0.05	1.00	−0.05	1.00	−0.01	1.00	−0.02	1.00	−0.03	–
Fisher alpha	0.96	0.25	0.97	0.08	0.96	0.22	0.98	0.16	0.98	0.13	0.97	0.17
Menhinick	0.96	0.24	0.97	0.07	0.96	0.23	0.98	0.16	0.98	0.14	0.97	0.17
Faith pd	0.95	0.16	0.95	0.03	0.76	0.05	0.95	0.13	0.98	0.08	0.92	0.09
Lladser pe	0.49	−0.09	0.57	−0.10	0.67	0.04	0.70	−0.14	0.55	−0.06	0.60	−0.07
Heip e	0.00	0.97	−0.11	0.91	0.07	0.91	−0.06	0.94	−0.04	0.92	−0.03	0.93
Simpson e	−0.22	0.89	−0.27	0.82	−0.05	0.86	−0.19	0.87	−0.10	0.85	−0.16	0.86
ENSpie	0.41	0.78	0.46	0.74	0.47	0.76	0.39	0.80	0.46	0.77	0.44	0.77
Strong	0.22	0.86	0.06	0.78	0.16	0.78	0.11	0.76	0.06	0.80	0.12	0.80
Shannon	0.51	0.76	0.66	0.66	0.52	0.73	0.58	0.73	0.64	0.68	0.58	0.71

### Richness and evenness were altered neither in Parkinson’s disease patients nor in MS patients in comparison with controls

**Figure 3:**
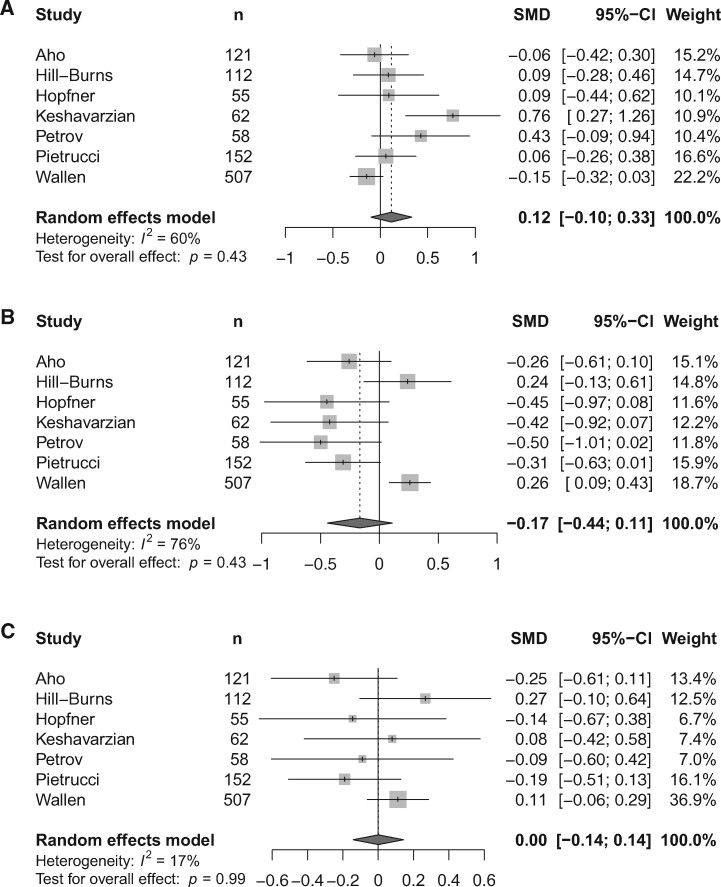
**Richness, evenness and Shannon index meta-analyses for Parkinson’s disease studies.** (**A**) Forest plot for the richness. (**B**) Forest plot for the evenness. (**C**) Forest plot for the Shannon index.

**Figure 4:**
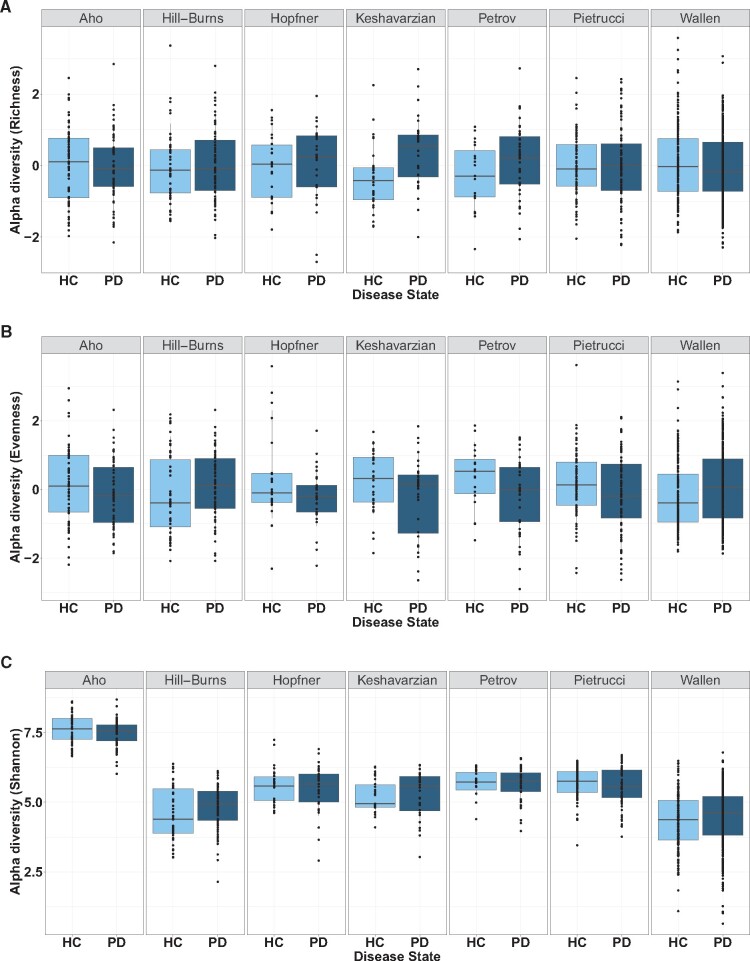
(**A**) Distribution of richness values for each study, stratified by disease groups (Parkinson’s disease vs HC). (**B**) Distribution of evenness values for each study, stratified by disease groups (Parkinson’s disease vs HC) (**C**) Distribution of Shannon values for each study, stratified by disease groups (Parkinson’s disease vs HC).

**Figure 5:**
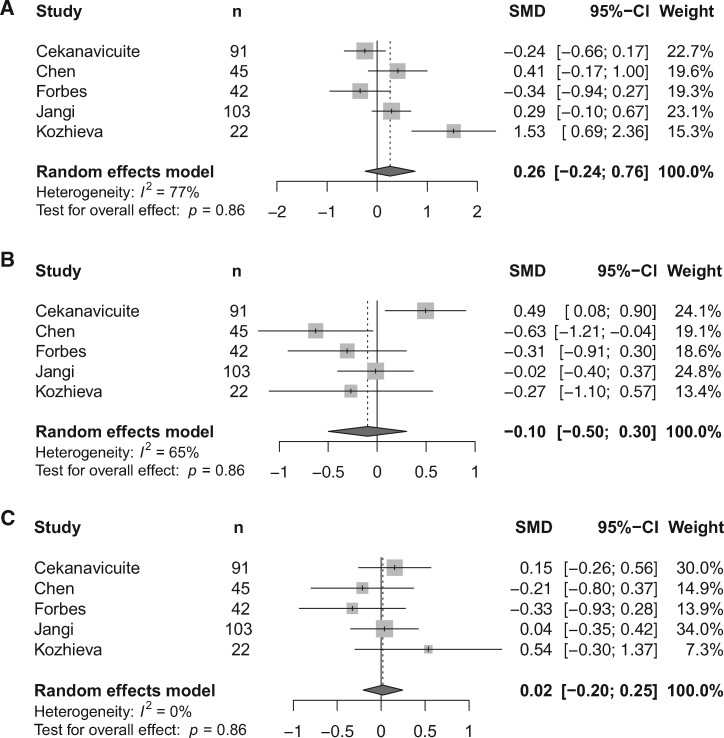
**Richness, evenness and Shannon index meta-analyses for Multiple Sclerosis studies.** (**A**) Forest plot for the richness. (**B**) Forest plot for the evenness. (**C**) Forest plot for the Shannon index.

**Figure 6:**
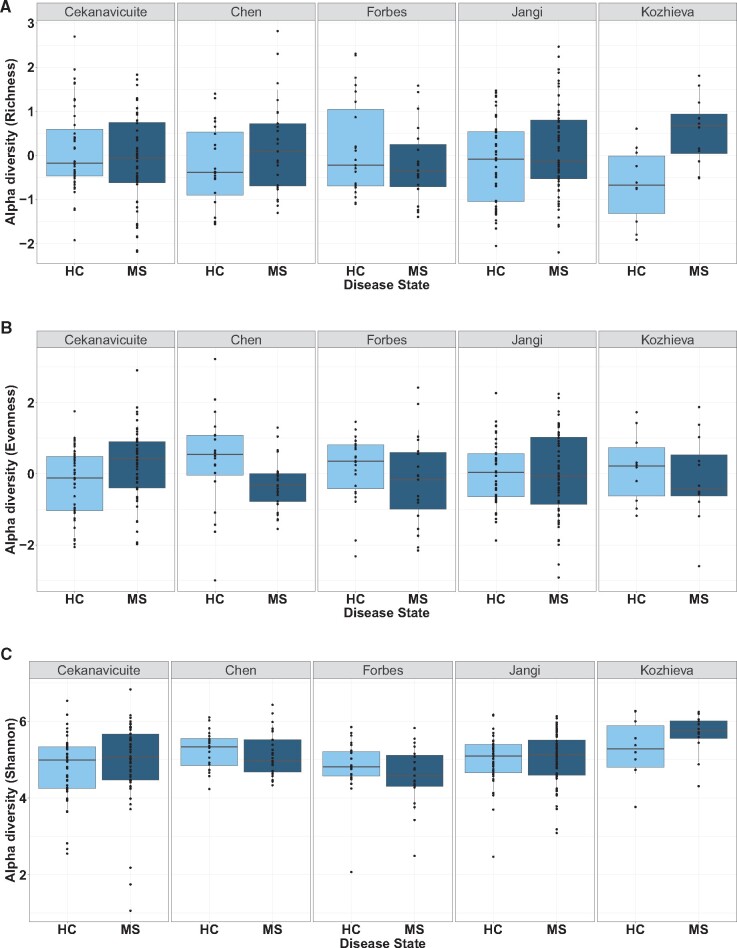
(**A**) Distribution of richness values for each study, stratified by disease groups (MS vs HC). (**B**) Distribution of evenness values for each study, stratified by disease groups (MS vs HC) (**C**) Distribution of Shannon values for each study, stratified by disease groups (MS vs HC).

For richness, we performed a weighting inverse variance meta-analysis with a random model. For each study, the SMD was estimated and reported on a Forest Plot with its confidence interval (see Fig. 3A for Parkinson’s disease studies, Fig. 5A for MS studies). We have also drawn distribution of diversity values for Parkinson’s disease studies and MS (respectively Figs 4A and 6A). The overall estimate was not significant for both diseases {overall estimate = 0.12 [95% CI (−0.10; 0.33)], adjusted *P*-value = 0.43 for Parkinson’s disease; overall estimate = 0.26 [95% CI (−0.24; 0.76)], adjusted *P*-value = 0.86 for MS}. The highest change in richness was observed for Keshavarzian et al., confirming results reported in this publication. We also adjusted SMD estimates for age and sex, two confounding factors. This adjustment was done for Parkinson’s disease studies only, but richness was still not altered in patients in comparison with donors {overall estimate = −0.02 [95% CI (−0.21; 0.18)], adjusted *P*-value = 0.87, Supplementary Fig. 2A}.

We performed the same analysis for evenness. Evenness was non-significantly decreased in Parkinson’s disease with an overall estimate at −0.17 [95% CI (−0.44; 0.11), adjusted *P*-value = 0.43], neither in MS {overall estimate = −0.10 [95% CI (−0.50; 0.30), adjusted *P*-value =0.86] [see Fig. 3B for Parkinson’s disease studies, Fig. 5B for MS studies]}. For Parkinson’s disease, the evenness was decreased in five studies, and increased in only two studies (Hill-Burns et al. and Wallen et al.). After adjustment for age and sex, the overall estimate was still not significant for Parkinson’s disease studies {overall estimate = −0.01 [95% CI (−0.26; 0.25)], adjusted *P*-value = 0.96, Supplementary Fig. 2B}, but the number of studies included into the meta-analysis was reduced to four.

### Shannon index was not associated neither to ND

We performed the same analysis on the Shannon index (see Fig. 3C for Parkinson’s disease studies, Fig. 5C for MS studies). The overall estimate was still not significant for both diseases {overall estimate = 0.00 [95% CI (−0.14; 0.14)], adjusted *P*-value =0.99 for Parkinson’s disease, overall estimate = 0.02 (95% CI (−0.20; 0.25)], adjusted *P*-value = 0.86 for MS}. For Parkinson’s disease studies, after adjustment for age and sex, the overall estimate was still not significant {overall estimate = −0.03 [95% CI (−0.11; 0.17)], adjusted *P*-value = 0.96, Supplementary Fig. 2C}.

## Discussion

Parkinson’s disease is the neurological disorder reporting the largest number of cross-sectional studies with targeted metagenomics,^54^ and results in α-diversity index were inconsistent. Shannon index was increased in Parkinson’s disease subjects in comparison with age-matched donors in various cross-sectional, observational studies,^19^^,^^20^^,^^40^ but in disagreement with others.^16^^,^^17^^,^^21^^,^^36^^,^^37^^,^^39^ The species richness (number of species or number of OTUs) differed also significantly in various studies,^19^^,^^40^ and sometimes in contradiction with Shannon results.^17^ Some studies reported α-diversity using Chao1, but results were contradictory. For example, Chao1 index was increased in Parkinson’s disease subjects in comparison with age-matched donors,^19^^,^^28^ in disagreement with others^21^^,^^36^ and in contradiction with one study observed where an increase of Chao1 in the donor group was observed.^18^ The changes in α-diversity between adults with MS compared to controls were unclear. Richness was increased in primary progressive MS patients in comparison to controls^29^ but not Chao1. Shannon index and species richness were significantly decreased in one study^27^ while only trends or non-significant results were reported on others. Regarding Alzheimer’s disease patients, two cross-sectional studies have reported alteration of α-diversity in comparison with healthy controls.^34^^,^^35^

One of the main objectives of our study was to analyse measures of α-diversity from ND studies using an empirical method to make studies comparable. We applied an EFA to each study in order to reduce the complexity and multiplicity of α-diversity indices. This method, described and justified by Hagerty et al., aims at estimating two robust and reliable α-diversity composite measures, richness and evenness, to simplify statistical association tests with clinical outcome and comparison between studies. Our results have revealed that the EFA is a powerful method for estimating richness and evenness by catching a large proportion of the variance into the two first factor loadings. We applied EFA independently to the six Parkinson’s disease studies and five MS studies, and we observed a strong consistency between all studies, meaning the correlation structure between α-diversity indices and the two composite factors was reproducible. Menhinick, Faith pd, Fisher alpha and Llader pe indices were all associated to richness, Simpson e, Heip e, ENSpie and Strong were all associated to evenness, while Shannon index was associated to both.

The link between α-diversity measures and richness or evenness factor loadings was quite different in comparison to Hagerty et al.,^46^ that reports Shannon and ENSpie both being associated to richness, while Lladser pe was associated to evenness. ENSpie, equivalent to the inverse Simpson index, can be interpreted as the number of equally abundant species in a sample, as such, it is a combination of richness and evenness. In our study, ENSpie was more related to the evenness, however, we also observed a weak association with the richness (correlation of 0.44) despite the orthogonality between factor loadings. Hagerty et al. associated Shannon to richness, but like ENSpie, and because Shannon directly depends on the number of species and their proportion, we think that we should not reduce both Shannon index and ENSpie to only one category, and their association with one or the other will strongly depend on the data and on the number of entities used for calculations. For Lladser pe, the difference in categorization can be explained by first the lower proportion of variance explained by both factors regarding this index but also by the nature of this index, measuring how much of the sample contains unsampled species.

By nature and operational construction, richness and evenness are orthogonal ^46^ meaning that they vary independently of each other. This is a key point because lot of studies reduce the analysis of α-diversity to the richness only, for example by reporting the number of observed OTUs. But evenness might be systematically reported for its complementarity to richness even if they are both related to the same species. We can easily imagine a simple case where two species disappear in a community A of hundreds of species and two new species appear in a community B, in conjunction with a strong disruption in abundances in only one of the two communities (for example, a blooming of some species). In that case, the richness will remain unchanged, while the evenness will be altered. Similarly in Parkinson’s disease, the meta-analysis revealed a small alteration of evenness while the richness was unchanged, this reflects changes in the abundance of some specific taxa.

Alpha-diversity is the most validated metagenomic marker of GI health and metabolic disorders. The loss of diversity, mainly measured with α-diversity indices, has been linked to severity of a multitude of diseases,^55^ such as Inflammatory Bowel Disease,^56^ obesity and metabolic syndromes^57^^,^^58^ or HIV.^59^ There is not yet a gold standard regarding α-diversity measures, even if the number of species (or Operational Taxonomic Units) and the Shannon diversity index are the two most widespread indices reported in the literature for ND studies. We evaluated whether richness, Shannon index and evenness were suitable markers of ND by performing an exhaustive re-analysis of published metagenomic datasets. Our aim was to evaluate the potential of these makers as diagnostic markers of ND. Richness and the Shannon index were associated neither with Parkinson’s disease, nor with MS. Evenness was not associated with MS, however, the meta-analysis revealed a trend for decreased evenness in Parkinson’s disease where the adjusted *P*-value was not significant but the 95% CI of the overall estimate did not include the zero value. This result should be interpreted with caution.

Our study has some limitations. First, the sample size of each study did not exceed 100 samples per group, with an exception for Parkinson’s disease studies with the inclusion a large cohort from Wallen et al. In biomarker research studies based on metagenomic datasets, it is advised to include more than 100 samples per group to increase power and to deal with inter-sample variability, which is stronger in metagenomics in comparison with other omics data. Secondly, our meta-analysis included various populations in Europe, USA and Japan. Various studies from Chinese patients have been published in Parkinson’s disease, but without access to the data we cannot conclude on α-diversity alteration for this population. Another limitation was the lack of clinical data, and therefore the impossibility to take into account potential cofounding factors. It is well-known that age, BMI, constipation and COMT treatment are the main confounding factors reported in the literature for their association with gut microbiota.^2^^,^^38^ For Parkinson’s disease, we have obtained some individual clinical data, mainly age and sex, that we adjusted standardized mean difference estimates for. Results for evenness, after adjusting, were still not significant, but the number of studies was reduced to four. Another limitation was the characterization of individual phenotypes and the absence of stratification regarding disease progression in the meta-analysis. This meta-analysis did not filter studies based on inclusion and exclusion criteria of each study; however, we know these clinical criteria can be strongly different from one study to another, and may affect consistency of findings when no adjustment for cofounding factors can be done.

In QIIME 2, one of the most popular pipelines for processing 16S rRNA sequencing files,^47^ no less than thirty-one diversity metrics are available. Selecting the most useful indices can be challenging. Many of these metrics are highly correlated like Chao1 and the number of OTUs, and wrong selection of indices to analyse can lead to misinterpretation and/or multiplicity issues with statistical tests. If some α-diversity indices are comprehensible and easy to interpret, like the number of species, others can be elaborate like Faith’s pd index which uses a phylogenetic tree. Then, α-diversity metrics are not directly comparable between independent studies, due to variability in sample processing (targeted region, DNA extraction kit, PCR amplification, sequencing device) and diverse bioinformatics pipelines. Even the same metric, such as Shannon diversity, can result in different values due to the bioinformatics packages implementing different log scales in the algorithm. The method proposed by Hagerty and colleagues facilitates both the analysis and the interpretation, but we also demonstrated that composite measures derived from this method are suitable for meta-analysis.

Today, loss of α-diversity is considered by many experts in the field as a major societal concern to the industrialized world and a potential cause for the increase in common diseases, such as inflammatory bowel disease, allergies, cancers, autism and metabolic syndrome. However, for it to be a useful clinical marker of human health, it is important to document specifically which diseases it is associated with and to what extent rebalancing the gut microbiome can be an effective public health prevention strategy in industrialized countries.

Decrease of α-diversity, and in particular the Shannon index, has been repeatedly reported to be associated to various diseases, and is therefore an important clinical marker of gut health. The diagnostic of loss of diversity can help to identify unhealthy subjects, but our results suggest that these diagnostic tests will not be suitable for Parkinson’s disease and MS if they limit their interpretation of gut microbiota to only α-diversity. We hypothesize that the ND gut microbiome has a more subtle dysbiosis than in GI diseases such as inflammatory bowel disease or metabolic disorders such as obesity and diabetes where observed differences in α-diversity are striking. It is likely that an increase/decrease in only a limited number of bacteria are involved in the disease aetiology.

Our study was limited to α-diversity measures available using the QIIME2 pipeline. However, other α-diversity indices were developed to account for the lack of variance estimates and lack of unobserved species in their estimation.^45^ It would be suitable to democratize the access to these α-diversity indices like the Chao-Bunge and breakaway^45^ throughout a unique platform and to go beyond the simple richness and evenness measures. Another initiative would to extend analysis of diversity analysis to functional diversity, and more precisely to functional divergence indicating a high degree of niche differentiation^60^ and analysis of diversification.^61^

## Supplementary material

Supplementary material is available at *Brain Communications* online.

## Supplementary Material

fcab113_Supplementary_Data

## Abbreviations

α-diversity =: alpha-diversity
EFA =: exploratory factor analysis
GI =: gastro-intestinal
MS =: multiple sclerosis
ND =: neurological disorders
SMD =: standardized mean differences
