# Supplementary material for: Gut microbiome alpha-diversity is not a marker of Parkinson’s disease and multiple sclerosis
Source: Brain Commun. 2021 Jun 1;3(2):fcab113. doi: 10.1093/braincomms/fcab113 (PMC8195527; doi:10.1093/braincomms/fcab113)
Supplement: fcab113_Supplementary_Data [file fcab113_supplementary_data.pdf]

Supplementary material

Supplementary Figure 1: Number of sequence counts for each study

A) Number of sequence counts for each PD study. Each point corresponds to one sample. The horizontal line corresponds to the threshold of 10 000 sequences under which the sample was removed. B) Number of sequence counts for each MS study. Each point corresponds to one sample. The horizontal line corresponds to the threshold of 10 000 sequences under which the sample was removed.

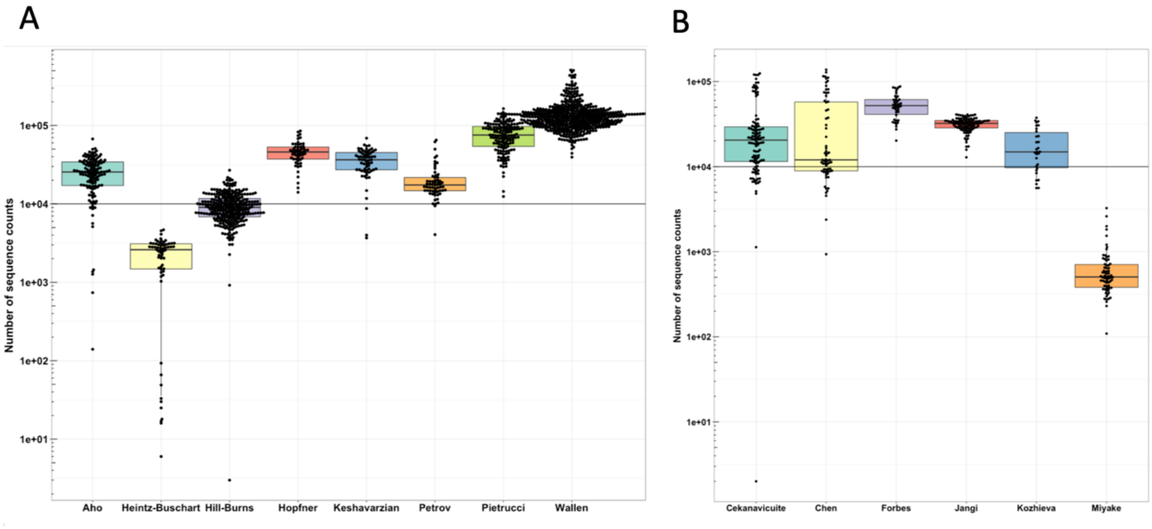

Supplementary Figure 2: Meta-analysis of richness, evenness and Shannon index for PD studies after adjusting for Age and Sex.

Richness, evenness and Shannon index meta-analyses for Parkinson's Disease studies. A) Forest plot for the richness. B) Forest plot for the evenness. C) Forest plot for the Shannon index.

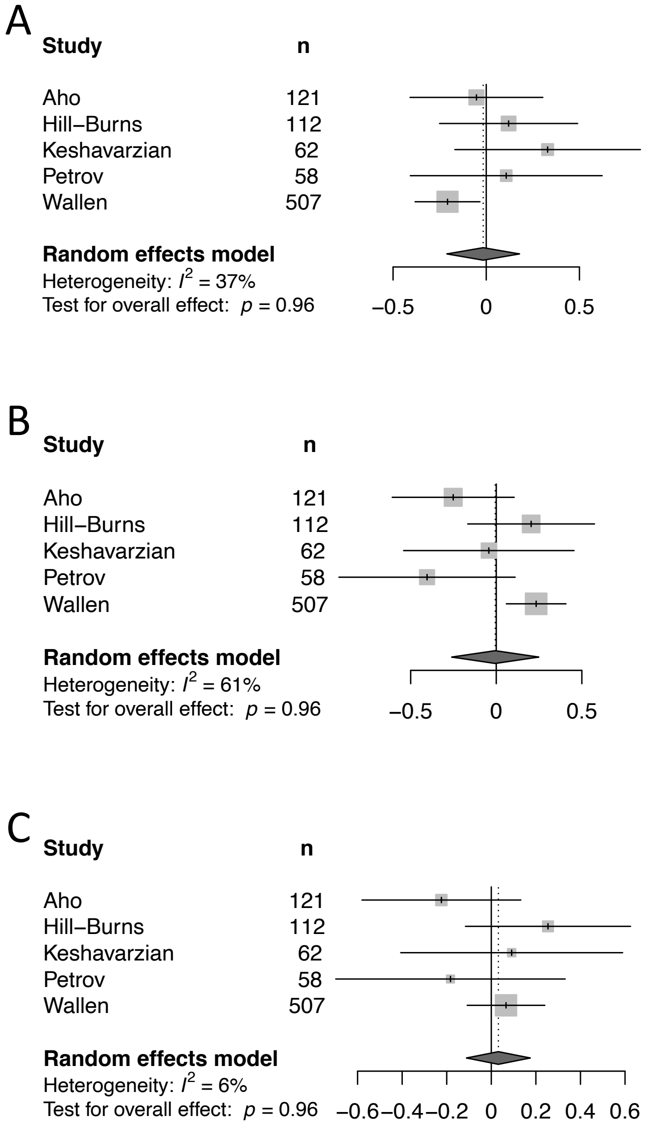

Supplementary Table 1: original studies from Query#1 (Parkinson's Disease) matching our selection criteria

	16S data	PD vs HC info	Clinical/metadata	Country	N (PD/HC)	Techno	16S region	Included into the meta-analysis?
**Keshavarzian *et al.*** [1]	SRR1734369	Yes	Yes	USA	34/31	Illumina	V4	Yes
**Hill-Burns *et al.*** [2]	ERP016332	Yes	Yes	USA	197/130	Illumina	V4	Yes
**Petrov *et al.*** [3]	Provided by Dr. Petrov	Yes	Yes	Russian	89/66	Illumina	V3-V4	Yes
**Hopfner *et al.*** [4]	PRJEB14928	Yes	No	Germany	29/29	Illumina	V1-V2	Yes
**Li W *et al.*** [5]	No	No	No	China	24/14	Illumina	V3-V4 + V4-V5	No
**Heintz-Buscart *et al.*** [6]	PRJNA381395	Yes	No	Germany	26/38	Illumina	V4	No*
**Qian *et al.*** [7]	PRJNA391524	No	No	China	45/45	Illumina	V3-V4	No
**Lin A *et al.*** [8]	No	No	No	China	75/45	Illumina	V4	No
**Barichella *et al.*** [9]	No	No	No	Italy	193/113	Illumina	V3-V4	No
**Pietrucci *et al.*** [10]	PRJNA510730	Yes	No	Italy	80/82	Illumina	V3-V4	Yes
**Li F *et al.*** [11]	No	No	No	China	10/10	Illumina	V3-V4	No
**Aho *et al.*** [12]	PRJEB27564	Yes	Yes	Finland	64/64	Illumina	V3-V4	Yes
**Lin CH *et al.***[13]	No	No	No	China	80/80	Illumina	V3-V4	No
**Li C *et al.***[14]	No	No	No	China	51/48	Illumina	V3-V4	No

**Vidal-Martinez *et al.***[15]	No access – Not approved by IRB	No	No	USA	10/10	Illumina	V3-V4	No
**Hegelmaier *et al.***[16]	No	No	No	German	54/32	Illumina	V1-V3	No
**Ren *et al.***[17]	PRJNA561023	No	No	China	27/13	Illumina	V4-V5	No
**Baldini *et al.***[18]	No	No	No	Lux/German	147/162	Illumina	V3-V4	No
**Wallen *et al.*** [19]	PRJNA601994 (but still not available by June 2020)	No	No	USA	323/184	Illumina	V4	No

(*) sequencing files were processed but the sequencing depth was too low for an accurate quantification of alpha-diversity measures.

Supplementary Table 2: original studies for Query#2 (Multiple Sclerosis Disease) matching our selection criteria

	16S data	MS vs HC info	Clinical/metadata	Country	N (MS/HC)	Techno	16S region	Included into the meta-analysis?
**Miyake *et al.*** [20]	For DDBJ accession numbers, see publication	Yes	No	Japan	20/58	Illumina	V1-V2	No*
**Chen *et al.*** [21]	Ask AC/MF	Yes	No	USA	31/36	Illumina	V3-V5	Yes
**Jangi *et al.*** [22]	PRJNA321051	Yes	No	Canada	60/43	Illumina	V4	Yes
**Cekanaviciute *et al.*** [23]	Ask AC/MF	Yes	No	USA	70/70	Illumina	V4	Yes
**Forbes *et al*.**[24]	PRJNA450340	Yes	No	Canada	19/23	Illumina	V4	Yes
**Kozhieva et al.** [25]	PRJNA565173	Yes	No	Russian	15/15	Illumina	V3-V4	Yes
**Reynders et al.** [26]	No	No	No	Belgian	98/120	Illumina	V4	No

(*) sequencing files were processed but the sequencing depth was too low for an accurate quantification of alpha-diversity measures.

Supplementary Table 3: original studies for Query#3 (Alzheimer's Disease) matching our selection criteria

	16S data	AD vs HC info	Clinical/metadata	Country	N (AD/HC)	Techno	16S region	Included into the meta-analysis?
**Vogt *et al.*** [27]	Under request	Yes	Under request	USA	25/25	Illumina	V4	No
**Zhuang et al.** [28]	No	No	No	China	43/43	Illumina	V3-V4	No

References

1. Keshavarzian A, Green SJ, Engen PA, u. a. Colonic bacterial composition in Parkinson's disease. *Mov Disord*. 2015;30(10):1351–1360. doi:10.1002/mds.26307
2. Hill-Burns EM, Debelius JW, Morton JT, u. a. Parkinson's disease and Parkinson's disease medications have distinct signatures of the gut microbiome. *Mov Disord*. 2017;32(5):739–749. doi:10.1002/mds.26942
3. Petrov VA, Saltykova I V., Zhukova IA, u. a. Analysis of gut microbiota in patients with parkinson's disease. *Bull Exp Biol Med*. 2017;162(6):734–737. doi:10.1007/s10517-017-3700-7
4. Hopfner F, Künstner A, Müller SH, u. a. Gut microbiota in Parkinson disease in a northern German cohort. *Brain Res*. 2017;1667:41–45. doi:10.1016/j.brainres.2017.04.019
5. Li W, Wu X, Hu X, u. a. Structural changes of gut microbiota in Parkinson's disease and its correlation with clinical features. *Sci China Life Sci*. 2017;60(11):1223–1233. doi:10.1007/s11427-016-9001-4
6. Heintz-Buschart A, Pandey U, Wicke T, u. a. The nasal and gut microbiome in Parkinson's disease and idiopathic rapid eye movement sleep behavior disorder. *Mov Disord*. 2018;33(1):88–98. doi:10.1002/mds.27105
7. Qian Y, Yang X, Xu S, u. a. Alteration of the fecal microbiota in Chinese patients with Parkinson's disease. *Brain Behav Immun*. 2018;70:194–202. doi:10.1016/j.bbi.2018.02.016
8. Lin A, Zheng W, He Y, u. a. Gut microbiota in patients with Parkinson's disease in southern China. *Park Relat Disord*. 2018;53(April):82–88.

doi:10.1016/j.parkreldis.2018.05.007

9. Barichella M, Severgnini M, Cilia R, u. a. Unraveling gut microbiota in Parkinson's disease and atypical parkinsonism. *Mov Disord*. 2019;34(3):396–405. doi:10.1002/mds.27581

10. Pietrucci D, Cerroni R, Unida V, u. a. Dysbiosis of gut microbiota in a selected population of Parkinson's patients. *Park Relat Disord*. 2019;65(June):124–130. doi:10.1016/j.parkreldis.2019.06.003

11. Li F, Wang P, Chen Z, Sui X, Xie X, Zhang J. Alteration of the fecal microbiota in North-Eastern Han Chinese population with sporadic Parkinson's disease. *Neurosci Lett*. 2019;707:134297. doi:10.1016/j.neulet.2019.134297

12. Aho VTE, Pereira PAB, Voutilainen S, u. a. Gut microbiota in Parkinson's disease: Temporal stability and relations to disease progression. *EBioMedicine*. 2019;44:691–707. doi:10.1016/j.ebiom.2019.05.064

13. Lin CH, Chen CC, Chiang HL, u. a. Altered gut microbiota and inflammatory cytokine responses in patients with Parkinson's disease. *J Neuroinflammation*. 2019;16(1):1–9. doi:10.1186/s12974-019-1528-y

14. Li C, Cui L, Yang Y, u. a. Gut microbiota differs between parkinson's disease patients and healthy controls in northeast China. *Front Mol Neurosci*. 2019;12(July):1–13. doi:10.3389/fnmol.2019.00171

15. Vidal-Martinez G, Chin B, Camarillo C, u. a. A Pilot Microbiota Study in Parkinson's Disease Patients versus Control Subjects, and Effects of FTY720 and FTY720-Mitoxy Therapies in Parkinsonian and Multiple System Atrophy Mouse Models. *J Parkinsons Dis*. 2020;10(1):185–192. doi:10.3233/JPD-191693

16. Hegelmaier T, Lebbing M, Duscha A, u. a. Interventional Influence of the Intestinal Microbiome Through Dietary Intervention and Bowel Cleansing Might Improve Motor Symptoms in Parkinson's Disease. *Cells*. 2020;9(2):376. doi:10.3390/cells9020376

17. Ren T, Gao Y, Qiu Y, u. a. Gut Microbiota Altered in Mild Cognitive Impairment Compared With Normal Cognition in Sporadic Parkinson's Disease. *Front Neurol*. 2020;11(February):1–8. doi:10.3389/fneur.2020.00137

18. Baldini F, Hertel J, Sandt E, u. a. Parkinson ' s disease-associated alterations of the gut microbiome can invoke disease-relevant metabolic changes. *bioRxiv*. Published online 2019:1–36.

19. Wallen ZD, Appah M, Dean MN, u. a. Characterizing dysbiosis of gut microbiome in PD: evidence for overabundance of opportunistic pathogens. *npj Park Dis*.

2020;6(1):1–12. doi:10.1038/s41531-020-0112-6

20. Miyake S, Kim S, Suda W, u. a. Dysbiosis in the gut microbiota of patients with multiple sclerosis, with a striking depletion of species belonging to clostridia XIVa and IV clusters. *PLoS One*. 2015;10(9):1–16. doi:10.1371/journal.pone.0137429
21. Chen J, Chia N, Kalari KR, u. a. Multiple sclerosis patients have a distinct gut microbiota compared to healthy controls. *Sci Rep*. 2016;6:1–10. doi:10.1038/srep28484
22. Jangi S, Gandhi R, Cox LM, u. a. Alterations of the human gut microbiome in multiple sclerosis. *Nat Commun*. 2016;7(May). doi:10.1038/ncomms12015
23. Cekanaviciute E, Yoo BB, Runia TF, u. a. Erratum: Gut bacteria from multiple sclerosis patients modulate human T cells and exacerbate symptoms in mouse models (Proc Natl Acad Sci USA (2017) 114 (10713–10718) DOI: 10.1073/pnas.1711235114). *Proc Natl Acad Sci U S A*. 2017;114(42):E8943. doi:10.1073/pnas.1716911114
24. Forbes JD, Chen CY, Knox NC, u. a. A comparative study of the gut microbiota in immune-mediated inflammatory diseases - Does a common dysbiosis exist? *Microbiome*. 2018;6(1):1–15. doi:10.1186/s40168-018-0603-4
25. Kozhieva M, Naumova N, Alikina T, Boyko A, Vlassov V, Kabilov MR. Primary progressive multiple sclerosis in a Russian cohort: Relationship with gut bacterial diversity. *BMC Microbiol*. 2019;19(1):1–9. doi:10.1186/s12866-019-1685-2
26. Reynders T, Devolder L, Valles-Colomer M, u. a. Gut microbiome variation is associated to Multiple Sclerosis phenotypic subtypes. *Ann Clin Transl Neurol*. Published online 2020:406–419. doi:10.1002/acn3.51004
27. Vogt NM, Kerby RL, Dill-McFarland KA, u. a. Gut microbiome alterations in Alzheimer's disease. *Sci Rep*. 2017;7(1):1–11. doi:10.1038/s41598-017-13601-y
28. Zhuang ZQ, Shen LL, Li WW, u. a. Gut Microbiota is Altered in Patients with Alzheimer's Disease. *J Alzheimer's Dis*. 2018;63(4):1337–1346. doi:10.3233/JAD-180176
